# Antimicrobial susceptibility of *Stenotrophomonas maltophilia* from United States medical centers (2019–2023)

**DOI:** 10.1128/aac.00124-25

**Published:** 2025-03-06

**Authors:** Helio S. Sader, Mariana Castanheira, S. J. Ryan Arends, Timothy B. Doyle

**Affiliations:** 1 Element Iowa City (JMI Laboratories) 138461 , North Liberty, Iowa, USA; Universita degli studi di roma La Sapienza, Rome, Italy

**Keywords:** aztreonam-avibactam, minocycline, antimicrobial resistance, pneumonia, hospital-acquired infection, beta-lactamase inhibitor combination

## Abstract

We evaluated the antimicrobial susceptibility of 1,400 clinical isolates of *Stenotrophomonas maltophilia* consecutively collected from United States medical centers in 2019–2023. Aztreonam-avibactam (MIC_50/90_, 2/4 µg/mL; 99.6% inhibited at ≤8 µg/mL) was the most active compound, followed by trimethoprim-sulfamethoxazole (MIC_50/90_, ≤0.12/0.5 µg/mL; 96.9% susceptible), minocycline (MIC_50/90_, 0.5/2 µg/mL; 89.2% susceptible), and levofloxacin (MIC_50/90_, 1/8 µg/mL; 78.9% susceptible). Aztreonam-avibactam retained potent activity against isolates not susceptible to trimethoprim-sulfamethoxazole, minocycline, and/or levofloxacin (99.3%–100.0% inhibited at ≤8 µg/mL).

## INTRODUCTION

The occurrence of *Stenotrophomonas maltophilia* infections increased markedly in the last years, and the antimicrobial treatment options remain very limited (1, 2). *S. maltophilia* is intrinsically resistant to most antimicrobial agents commonly used to treat hospital-acquired gram-negative infections, including the recently approved β-lactamase inhibitor combinations ceftazidime-avibactam, meropenem-vaborbactam, and imipenem-relebactam (3, 4). Trimethoprim-sulfamethoxazole (TMP-SMX) is usually the preferred agent due to consistent *in vitro* activity and extensive clinical experience; however, there are very limited data from randomized clinical trials supporting the use of TMP-SMX for severe *S. maltophilia* infections (5–7). Fluoroquinolones, mainly levofloxacin, have also been used, and minocycline has recently emerged as a potential option, but there is limited information on the clinical efficacy of these compounds (5–7).

Aztreonam-avibactam was approved in April 2024 by the European Medicines Agency in the European Union (https://www.ema.europa.eu/en/news/new-antibiotic-fight-infections-caused-multidrug-resistant-bacteria; accessed on 7 October 2024) for treatment of adults with complicated intra-abdominal infection, hospital-acquired pneumonia, ventilator-associated pneumonia, and complicated urinary tract infection, and for the treatment of infections due to aerobic gram-negative bacteria for which there are limited treatment options, and it is under evaluation by the United States (US) Food and Drug Administration. Aztreonam is not hydrolyzed by metallo-β-lactamases (MBLs), and avibactam inhibits serine β-lactamases; thus, the aztreonam-avibactam combination is stable against hydrolysis by both L1 (an MBL) and L2 (serine carbapenemase) produced by *S. maltophilia* (8). In the present study, we evaluated the *in vitro* activities of aztreonam-avibactam and comparators against a large collection of *S. maltophilia* from US medical centers.

A total of 1,400 clinical isolates of *S. maltophilia* were consecutively collected from 62 US medical centers in 2019–2023 via the INFORM Program (9). Infection sites included pneumonia (*n* = 1,020; 72.9%), skin and skin structure infection (SSSI; *n* = 124; 8.9%), bloodstream infection (BSI; *n* = 117; 8.4%), urinary tract infection (UTI; *n* = 48; 3.4%), intra-abdominal infection (*n* = 30; 2.1%), and others (*n* = 61; 4.4%). Only isolates determined to be clinically significant by local criteria as the reported likely cause of infection were included in the investigation. Susceptibility testing was performed by Clinical and Laboratory Standards Institute (CLSI) broth microdilution methods at a monitoring laboratory (Element Iowa City [JMI Laboratories], North Liberty, IA, USA) (10). Aztreonam-avibactam was tested with avibactam at fixed 4 µg/mL and a pharmacodynamic/pharmacokinetic-susceptible breakpoint of ≤8 µg/mL applied for comparison (11).

**Fig 1:**
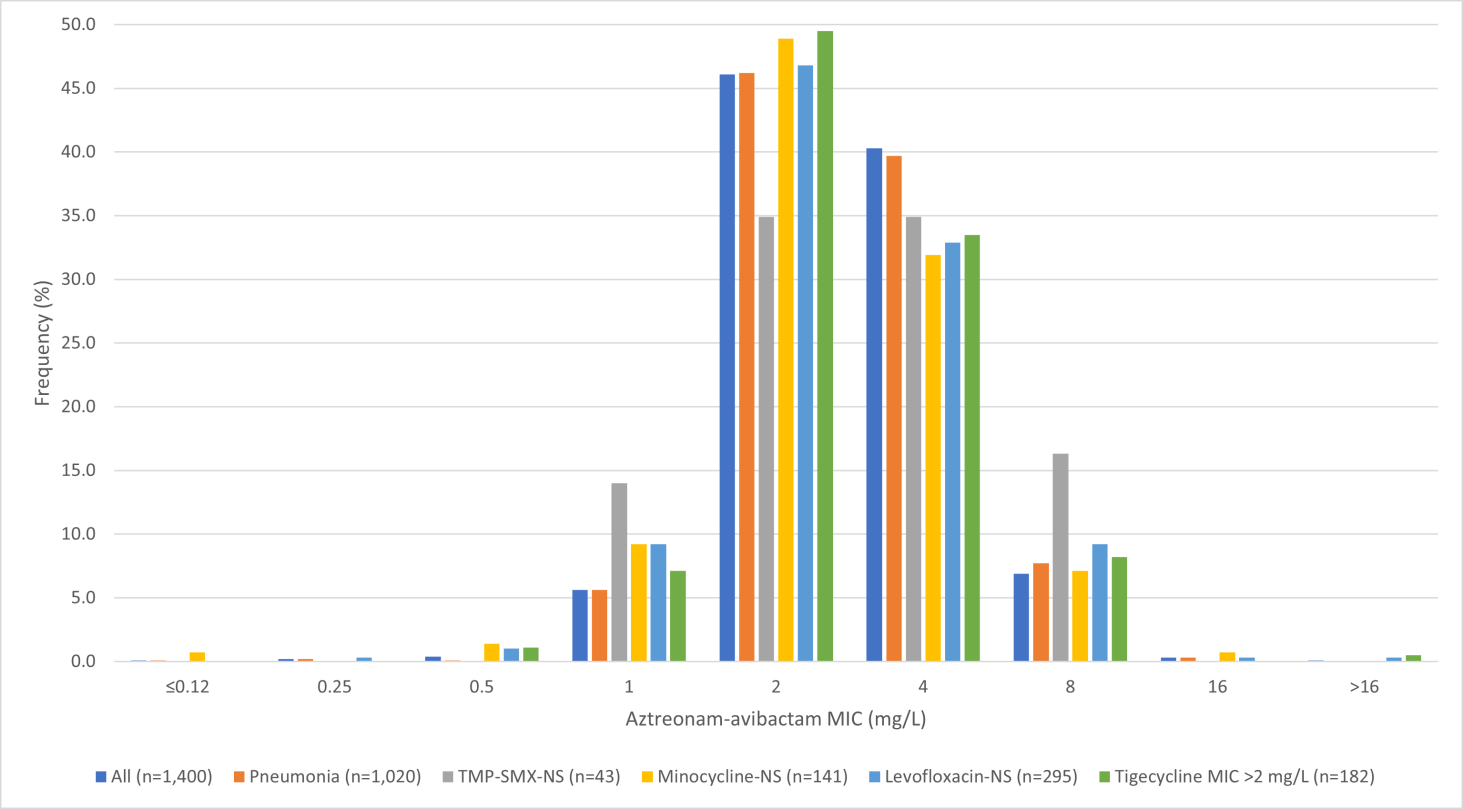
Aztreonam-avibactam MIC distributions for *S. maltophilia* isolates. Abbreviations: TMP-SMX, trimethoprim-sulfamethoxazole; NS, nonsusceptible.

Aztreonam-avibactam inhibited 99.6% of isolates at ≤8 µg/mL (MIC_50/90_, 2/4 µg/mL) and demonstrated potent activity against isolates from all infection types (Table 1; Table S1). Aztreonam-avibactam inhibited 99.7% of isolates from pneumonia, 99.2% of isolates from SSSI, and 100.0% of isolates from BSI and UTI at ≤8 µg/mL (Table 1; Table S1). Furthermore, aztreonam-avibactam retained potent activity against isolates not susceptible to other agents commonly used to treat *S. maltophilia* infections, such as TMP-SMX (*n* = 43; MIC_50/90_, 4/8 µg/mL; 100.0% inhibited at ≤8 µg/mL), minocycline (*n* = 141; MIC_50/90_, 2/4 µg/mL; 99.3% inhibited at ≤8 µg/mL), levofloxacin (*n* = 295; MIC_50/90_, 2/4 µg/mL; 99.3% inhibited at ≤8 µg/mL), and tigecycline (*n* = 182; MIC_50/90_, 2/4 µg/mL; 99.5% inhibited at ≤8 µg/mL) (Table 1; Fig. 1).

**TABLE 1:** Activity of aztreonam-avibactam and comparator agents stratified by infection type and resistant subsets^*e*^

Infection site (no. isolates)	% Susceptible per 2024 CLSI criteria^*a*^				
	ATM-AVI^*b*^	TMP-SMX	Minocycline	Levofloxacin	Tigecycline^*c*^
All isolates (1,400)	99.6	96.9	89.2	78.9	87.0
Infection type					
Pneumonia (1,020)	99.7	96.6	89.0	78.6	87.2
SSSI (124)	99.2	97.6	89.4	79.0	89.5
BSI (117)	100.0	99.1	88.8	82.1	85.5
UTI (48)	100.0	100.0	91.9	83.3	89.6
IAI (30)	96.7	93.3	81.5	66.7	66.7
Other infections (61)	100.0	96.7	93.1	80.3	90.2
Resistant subsets					
TMP-SMX-NS (43)^*d*^	100.0	0.0	51.2	23.3	60.5
Minocycline-NS (141)^*d*^	99.3	85.8	0.0	15.6	19.9
Levofloxacin-NS (295)^*d*^	99.3	88.8	55.6	0.0	47.8
Tigecycline MIC >2 µg/mL (182)	99.5	90.7	31.1	15.4	0.0

^*a*^ MIC_50_, MIC_90_, and MIC range values for each antimicrobial agent are shown in Table S1.

^*b*^ Percent inhibited at ≤8 µg/mL for comparison purposes.

^*c*^ Percent inhibited at ≤2 µg/mL, which is the US FDA-susceptible breakpoint for Enterobacterales, for comparison.

^*d*^ Isolates not susceptible per CLSI M100 (2024) criteria.

^*e*^ ATM-AVI, aztreonam-avibactam; TMP-SMX, trimethoprim-sulfamethoxazole; SSSI, skin and skin structure infection; BSI, bloodstream infection; UTI, urinary tract infection; IAI, intra-abdominal infection; NS, nonsusceptible.

Only five isolates displayed aztreonam-avibactam MIC >8 µg/mL, four isolates at 16 µg/mL, and one isolate at >16 µg/mL. The isolates were collected in 2023 (three isolates), 2022 (one), and 2021 (one) from five different states (Kentucky, Massachusetts, Nebraska, Ohio, and Virginia) and from patients with pneumonia (three isolates), IAI (one), and SSSI (one). All five isolates were susceptible to TMP-SMX and minocycline, four were inhibited at tigecycline MIC ≤2 µg/mL, and three were susceptible to levofloxacin.

The most active comparator agents were TMP-SMX (MIC_50/90_, ≤0.12/0.5 µg/mL; 96.9% susceptible) and minocycline (MIC_50/90_, 0.5/2 µg/mL; 89.2% susceptible; Table 1). Susceptibility to TMP-SMX per CLSI criteria (≤2 µg/mL) was lower among isolates not susceptible to minocycline (85.8% susceptible), levofloxacin (88.8% susceptible), or tigecycline (90.7% susceptible) compared to the overall collection (96.9% susceptible; Table 1). Notably, minocycline was active against only 51.2% of TMP-SMX–nonsusceptible isolates, 55.6% of levofloxacin-nonsusceptible isolates, and 31.1% of isolates with tigecycline MICs > 2 µg/mL (Table 1).

The fluoroquinolones exhibited modest activity against *S. maltophilia*. Levofloxacin (MIC_50/90_, 1/8 µg/mL) inhibited 78.9% of isolates at the current CLSI-susceptible breakpoint of ≤2 µg/mL (Table 1), whereas ciprofloxacin (MIC_50/90_, 2/>4 µg/mL) inhibited only 3.7% of isolates at ≤0.5 µg/mL, which is the current CLSI breakpoint for *Pseudomonas aeruginosa* (data not shown). Moxifloxacin was the most active (lowest MIC values) fluoroquinolone with MIC_50/90_ values of 0.5/4 µg/mL. Moxifloxacin inhibited 79.7% of isolates at ≤1 µg/mL and 43.0% of isolates at ≤0.25 µg/mL, which is the current European Committee on Antimicrobial Susceptibility Testing (EUCAST) breakpoint for Enterobacterales (data not shown) (12).

The evaluation of tigecycline *in vitro* activity varies markedly depending on the breakpoint applied. Overall, 87.0% of isolates were inhibited at ≤2 µg/mL (Table 1), which is the susceptible breakpoint published by the US FDA for Enterobacterales (www.fda.gov/drugs/development-resources/antibacterial-susceptibility-test-interpretive-criteria). However, only 37.7% of isolates were inhibited at ≤0.5 µg/mL, which is the EUCAST breakpoint for *Escherichia coli* and *Citrobacter koseri* (12). Colistin (MIC_50/90_, 8/>8 µg/mL; 34.9% inhibited at ≤2 µg/mL) and ceftazidime (MIC_50/90_, >32/>32 µg/mL; 22.2% inhibited at ≤8 µg/mL) showed reduced activity against *S. maltophilia* (data not shown).

We evaluated the antimicrobial susceptibility of a large collection of clinical isolates of *S. maltophilia* from US hospitals. Our results indicated that based on currently available susceptible breakpoints, aztreonam-avibactam was the most active compound and demonstrated broader *in vitro* coverage than TMP-SMX and minocycline. One limitation of this investigation is the absence of cefiderocol as a comparator. Cefiderocol has shown good *in vitro* activity against *S. maltophilia* with favorable clinical response in a few case reports; however, data supporting the use of cefiderocol for the treatment of *S. maltophilia* infections are still very limited (4, 13).

In summary, aztreonam-avibactam exhibited potent activity and broad coverage against *S. maltophilia* from US hospitals, and its activity was not adversely affected by resistance to other agents. Our results indicated that aztreonam-avibactam may represent a valuable option to treat *S. maltophilia* infections, addressing a major unmet medical need. Clinical studies are urgently warranted to evaluate the efficacy of aztreonam-avibactam against infection caused by *S. maltophilia*.

## SUPPLEMENTAL MATERIAL

Table S1 aac.00124-25-s0001.docx Activity of aztreonam-avibactam and comparator agents stratified by infection type and resistant subsets.

ASM does not own the copyrights to Supplemental Material that may be linked to, or accessed through, an article. The authors have granted ASM a non-exclusive, world-wide license to publish the Supplemental Material files. Please contact the corresponding author directly for reuse.
