# Supplementary material for: Antimicrobial susceptibility of Stenotrophomonas maltophilia from United States medical centers (2019–2023)
Source: Antimicrob Agents Chemother. 2025 Mar 6;69(4):e00124-25. doi: 10.1128/aac.00124-25 (PMC11963544; doi:10.1128/aac.00124-25)
Supplement: Table S1 — Activity of aztreonam-avibactam and comparator agents stratified by infection type and resistant subsets. [file aac.00124-25-s0001.docx]

Table S1 Activity of aztreonam-avibactam and comparator agents stratified by infection type and resistant subsets.

| **Antimicrobial agent** | **MIC (µg/mL)** | | |  | **Susceptibility per CLSI ^a^** | | |  |
| --- | --- | --- | --- | --- | --- | --- | --- | --- |
|  | **50%** | **90%** | **Range** |  | **%S** | **%I** | **%R** |  |
| All isolates (1,400) |  |  |  |  |  |  |  |  |
| Aztreonam-avibactam | 2 | 4 | ≤0.03 to >16 |  | 99.6 |  |  |  |
| TMP-SMX | ≤0.12 | 0.5 | ≤0.12 to >4 |  | 96.9 |  | 3.1 |  |
| Minocycline | 0.5 | 2 | ≤0.06 to 16 |  | 89.2 | 7.6 | 3.2 |  |
| Levofloxacin | 1 | 8 | ≤0.015 to >32 |  | 78.9 | 9.3 | 11.8 |  |
| Tigecycline | 1 | 4 | ≤0.06 to >8 |  | 87.0 ^b^ |  |  |  |
| Pneumonia (1,020) |  |  |  |  |  |  |  |  |
| Aztreonam-avibactam | 2 | 4 | ≤0.03 to 16 |  | 99.7 |  |  |  |
| TMP-SMX | ≤0.12 | 1 | ≤0.12 to >4 |  | 96.6 |  | 3.4 |  |
| Minocycline | 0.5 | 2 | ≤0.06 to 8 |  | 89.0 | 7.5 | 3.5 |  |
| Levofloxacin | 1 | 8 | ≤0.015 to >32 |  | 78.6 | 9.6 | 11.8 |  |
| Tigecycline | 1 | 4 | 0.12 to >8 |  | 87.2 ^b^ |  |  |  |
| SSSI (124) |  |  |  |  |  |  |  |  |
| Aztreonam-avibactam | 2 | 4 | 0.5 to 16 |  | 99.2 |  |  |  |
| TMP-SMX | ≤0.12 | 0.5 | ≤0.12 to >4 |  | 97.6 |  | 2.4 |  |
| Minocycline | 0.25 | 2 | 0.12 to 16 |  | 89.4 | 8.9 | 1.6 |  |
| Levofloxacin | 1 | 8 | 0.12 to >32 |  | 79.0 | 10.5 | 10.5 |  |
| Tigecycline | 1 | 4 | 0.12 to 8 |  | 89.5 ^b^ |  |  |  |
| BSI (117) |  |  |  |  |  |  |  |  |
| Aztreonam-avibactam | 4 | 4 | 0.5 to 8 |  | 100.0 |  |  |  |
| TMP-SMX | ≤0.12 | 0.5 | ≤0.12 to >4 |  | 99.1 |  | 0.9 |  |
| Minocycline | 0.5 | 2 | 0.12 to 4 |  | 88.8 | 7.5 | 3.7 |  |
| Levofloxacin | 1 | 8 | 0.25 to 32 |  | 82.1 | 7.7 | 10.3 |  |
| Tigecycline | 1 | 4 | ≤0.06 to >8 |  | 85.5 ^b^ |  |  |  |
| UTI (48) |  |  |  |  |  |  |  |  |
| Aztreonam-avibactam | 2 | 4 | 1 to 8 |  | 100.0 |  |  |  |
| TMP-SMX | ≤0.12 | 0.5 | ≤0.12 to 2 |  | 100.0 |  | 0.0 |  |
| Minocycline | 0.5 | 1 | 0.12 to 2 |  | 91.9 | 8.1 | 0.0 |  |
| Levofloxacin | 1 | 8 | 0.25 to 32 |  | 83.3 | 6.2 | 10.4 |  |
| Tigecycline | 1 | 4 | 0.12 to 4 |  | 89.6 ^b^ |  |  |  |
| IAI (30) |  |  |  |  |  |  |  |  |
| Aztreonam-avibactam | 2 | 4 | 1 to >16 |  | 96.7 |  |  |  |
| TMP-SMX | 0.25 | 1 | ≤0.12 to 4 |  | 93.3 |  | 6.7 |  |
| Minocycline | 1 | 2 | 0.25 to 4 |  | 81.5 | 11.1 | 7.4 |  |
| Levofloxacin | 1 | 8 | 0.25 to 16 |  | 66.7 | 3.3 | 30.0 |  |
| Tigecycline | 2 | 4 | 0.25 to >8 |  | 66.7 ^b^ |  |  |  |
| Other infections (61) |  |  |  |  |  |  |  |  |
| Aztreonam-avibactam | 2 | 4 | 0.5 to 8 |  | 100.0 |  |  |  |
| TMP-SMX | 0.25 | 0.5 | ≤0.12 to >4 |  | 96.7 |  | 3.3 |  |
| Minocycline | 0.5 | 1 | 0.12 to 4 |  | 93.1 | 5.2 | 1.7 |  |
| Levofloxacin | 1 | 4 | 0.25 to >32 |  | 80.3 | 9.8 | 9.8 |  |
| Tigecycline | 1 | 2 | 0.12 to 4 |  | 90.2 ^b^ |  |  |  |
| TMP-SMX-NS (43) ^c^ |  |  |  |  |  |  |  |  |
| Aztreonam-avibactam | 4 | 8 | 1 to 8 |  | 100.0 |  |  |  |
| TMP-SMX | >4 | >4 | 4 to >4 |  | 0.0 |  | 100.0 |  |
| Minocycline | 1 | 4 | 0.12 to 16 |  | 51.2 | 24.4 | 24.4 |  |
| Levofloxacin | 16 | 32 | 0.25 to >32 |  | 23.3 | 7.0 | 69.8 |  |
| Tigecycline | 2 | 8 | 0.25 to >8 |  | 60.5 ^b^ |  |  |  |
| Minocycline-NS (141) ^c^ |  |  |  |  |  |  |  |  |
| Aztreonam-avibactam | 2 | 4 | 0.12 to 16 |  | 99.3 |  |  |  |
| TMP-SMX | 0.5 | 4 | ≤0.12 to >4 |  | 85.8 |  | 14.2 |  |
| Minocycline | 2 | 4 | 2 to 16 |  | 0.0 | 70.2 | 29.8 |  |
| Levofloxacin | 8 | 32 | 0.06 to >32 |  | 15.6 | 17.0 | 67.4 |  |
| Tigecycline | 4 | 8 | 0.5 to >8 |  | 19.9 ^b^ |  |  |  |
| Levofloxacin-NS (295) ^c^ |  |  |  |  |  |  |  |  |
| Aztreonam-avibactam | 2 | 4 | 0.25 to >16 |  | 99.3 |  |  |  |
| TMP-SMX | 0.5 | 4 | ≤0.12 to >4 |  | 88.8 |  | 11.2 |  |
| Minocycline | 1 | 4 | 0.12 to 16 |  | 55.6 | 29.5 | 14.9 |  |
| Levofloxacin | 8 | 16 | 4 to >32 |  | 0.0 | 44.1 | 55.9 |  |
| Tigecycline | 4 | 8 | 0.25 to >8 |  | 47.8 ^b^ |  |  |  |
| Tigecycline MIC >2 µg/mL (182) |  |  |  |  |  |  |  |  |
| Aztreonam-avibactam | 2 | 4 | 0.5 to >16 |  | 99.5 |  |  |  |
| TMP-SMX | 0.5 | 2 | ≤0.12 to >4 |  | 90.7 |  | 9.3 |  |
| Minocycline | 2 | 4 | 0.25 to 16 |  | 31.1 | 45.7 | 23.2 |  |
| Levofloxacin | 8 | 16 | 0.5 to >32 |  | 15.4 | 24.7 | 59.9 |  |
| Tigecycline | 4 | 8 | 4 to >8 |  | 0.0 ^b^ |  |  |  |

^a^ Criteria as published by CLSI M100 (2024) unless noted.
^b^ % inhibited at ≤2 µg/mL; which is the US FDA susceptible breakpoint for Enterobacterales for comparison.

^c^ Isolates not susceptible per CLSI M100 (2024) criteria.

Abbreviations: ATM-AVI, aztreonam-avibactam; TMP-SMX, trimethoprim-sulfamethoxazole; SSSI, skin and skin-structure infection; BSI, bloodstream infection; UTI, urinary tract infection; IAI, intra-abdominal infection; NS, nonsusceptible.
